# Full Van‐der‐Waals Graphene/h‐BN Hall Bars With Thickness‐Tuned Dielectric Shielding Enable Phase‐Coherent Spin Transport

**DOI:** 10.1002/smtd.202502182

**Published:** 2026-05-26

**Authors:** Feiyuan Ding, Kezhou Yang

**Affiliations:** ^1^ The Hong Kong University of Science and Technology – Guangzhou Campus Guangzhou Guangdong China

**Keywords:** dielectric shielding, full vdW heterostructures, graphene, quantum coherence, spin transport

## Abstract

Graphene/hexagonal Boron Nitride (h‐BN) devices offer high spin mobilities and atomically clean interfaces, yet spin coherence remains limited by inevitable interfacial imperfection and inseparable thickness‐scattering correlations. In this work, employing a full van‐der‐Waals (vdW) fabrication strategy, we fabricated graphene Hall bars on 11‐ and 22 nm h‐BN substrates, strategically selected to bracket the continuum dielectric regime where thickness‐tuned screening dominates. The devices exhibit sub‐ångström flatness and are devoid of interfacial bubbles, convinced by optical and scanning electron microscopy images. Magneto‐transport measurements show that such clean interfaces enable phase‐coherence lengths that approach the channel width, and meanwhile decouple spin transport characteristics from h‐BN dielectric thickness. First‐principles calculations confirm that interfacial charge transfer and proximity spin–orbit coupling saturate at the first h‐BN monolayer. Additional thickness merely enhances electrostatic screening without introducing disorder. This full vdW strategy delivers a scalable, back‐end‐compatible materials platform for ultra‐low‐dissipation two‐dimensional spin logic and topological superconducting circuits with atomically perfect interfaces.

## Graphical abstract

Full van der Waals graphene/h‐BN Hall bars with atomically perfect interfaces enable thickness‐tuned dielectric shielding. Varying h‐BN thickness (11 and 22 nm) switches quantum interference between weak anti‐localization and weak localization, achieving phase‐coherence lengths enhancement while maintaining spin coherence.

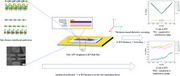

## 1 Introduction

Overcoming extrinsic disorder and the weak intrinsic spin–orbit coupling that presently restricts room‐temperature spin propagation in graphene is imperative for turning the material's record‐long spin diffusion lengths into scalable spin‐logic technologies [1]. It was found that atomically‐flat graphene/h‐BN interfaces eradicate the interfacial roughness and charge puddles that extrinsically limit spin lifetime, while moiré‐engineered proximity simultaneously amplifies spin–orbit coupling by an order of magnitude, directly addressing both disorder and weak‐ spinorbit coupling (SOC) constraints [2, 3]. These attributes transform graphene/h‐BN heterostructures into a tunable laboratory for exploring spin–valley‐layer locking, gate‐controllable topological edge states, and proximity‐induced magnetism or superconductivity, while remaining compatible with back‐end‐of‐line complementary metal‐oxide‐semiconductor (CMOS) processing [4]. Consequently, the system offers a rare combination of ballistic spin channels, electrically reconfigurable spin textures, and van‐der‐Waals integration, positioning it as a leading candidate for scalable, ultralow‐dissipation spin logic and memory.

However, in such graphene/h‐BN system, interfacial imperfections hinder the research on intrinsic SOC. For example, van‐der‐Waals assembling of graphene/h‐BN system will introduce sub‐micrometer hydrocarbon bubbles. The bubbles introduce local strain gradients and p–n junctions that curtail the spin lifetime from the intrinsic ∼100 to ≤1 ns [5]. Apart from bubbles, there may also be reactive graphene edges exposed during one‐dimensional contact formation. The exposed edges undergo sub‐nanometer roughening and oxidation, which generates random spin–orbit modulations that reduce spin‐injection polarization to <20% [6]. The interfacial imperfections also involve residual twist‐angle disorder (<0.5°), which further imposes a spatially varying proximity‐induced spin–orbit coupling of ±3 meV [7]. Such an effect invalidates homogeneous spin‐relaxation models and obscures the quantitative extraction of spin‐diffusion parameters [4].

To solve the interfacial imperfection bottleneck, recently, researchers proposed a full vdW strategy to fabricate the whole devices [8]. Conventionally, the vdW interfaces only exist in the graphene/h‐BN heterostructures. Meanwhile, the electrodes are usually fabricated by Laser direct writing or Electron beam lithography. As a result, this process is likely to destroy both the electrode/graphene interfaces and graphene/h‐BN interfaces due to the atom bombardment. In contrast, in the full vdW fabrication process, the pre‐patterned ferromagnetic electrode is mechanically transferred on exfoliated heterostructures, therefore converting the contact step from a symmetry‐breaking, contamination‐prone process into a deterministic van‐der‐Waals lamination that leaves both the graphene lattice and its buried h‐BN environment atomically unperturbed [8]. Since during the process graphene is never subjected to lithographic chemicals or reactive‐ion exposure, this full vdW fabrication strategy has the following advantages: (1) eliminates sub‐nanometer edge oxidation and random SOC modulation, (2) prevents hydrocarbon bubble entrapment by sealing the upper interface before ambient exposure [1], which suppresses intervalley scattering and preserves spin lifetimes exceeding 12 ns at room temperature [5], and (3) preserves the as‐exfoliated twist‐angle uniformity, enabling quantitative extraction of proximity‐enhanced SOC without spatial averaging [9]. This vdW strategy enables seamless, edge‐free geometry, which yields transparent, low‐impedance contacts (<100 Ω·µm) and spin‐injection polarizations above 90% while remaining fully compatible with back‐end‐of‐line CMOS processing [10].

In this work, by integrating mechanical exfoliation, deterministic dry‐transfer, and electrode lamination, we realize a graphene‐on‐h‐BN Hall bar in which both the buried h‐BN/graphene interface and the metal/graphene contact remain untouched by solvents, polymers, or reactive‐ion bombardment. We show that our full vdW fabrication strategy has the advantages of providing atomically flat interfaces without imperfections, and preventing hydrocarbon bubbles. Such interfacial perfection enhances the phase‐coherence length (l_φ_) to approach the device width and enables modulation of spin transport characteristics by changing the strength of the dielectric shielding effect without destructing any interface. By strategically selecting 11 and 22 nm h‐BN—spanning the transition from partial to full dielectric screening—we demonstrate that interfacial perfection enhances phase‐coherence length (L_φ_) to approach the device width, enabling thickness‐modulated spin transport governed purely by electrostatics rather than interfacial chemistry. Density‐functional theory calculations intentionally focus on dielectric response rather than spin‐orbit coupling, leveraging the experimentally established fact that proximity‐induced SOC saturates at the first h‐BN monolayer [2, 7], allowing us to isolate dielectric screening as the sole thickness‐dependent variable.

We further confirm that the perfect spin transport characteristics observed in the experimental measurement are due to the interfacial perfection by the Density Functional Theory (DFT) calculations. The dry‐lamination flow in the full vdW fabrication process is fully compatible with temperatures in back‐end‐of‐line CMOS fabrication processes, offering a realistic route toward ultralow‐dissipation spin logic, non‐local spin valves, and topological superconducting circuits at the two‐dimensional limit.

## 2 Discussion

**FIGURE 1:**
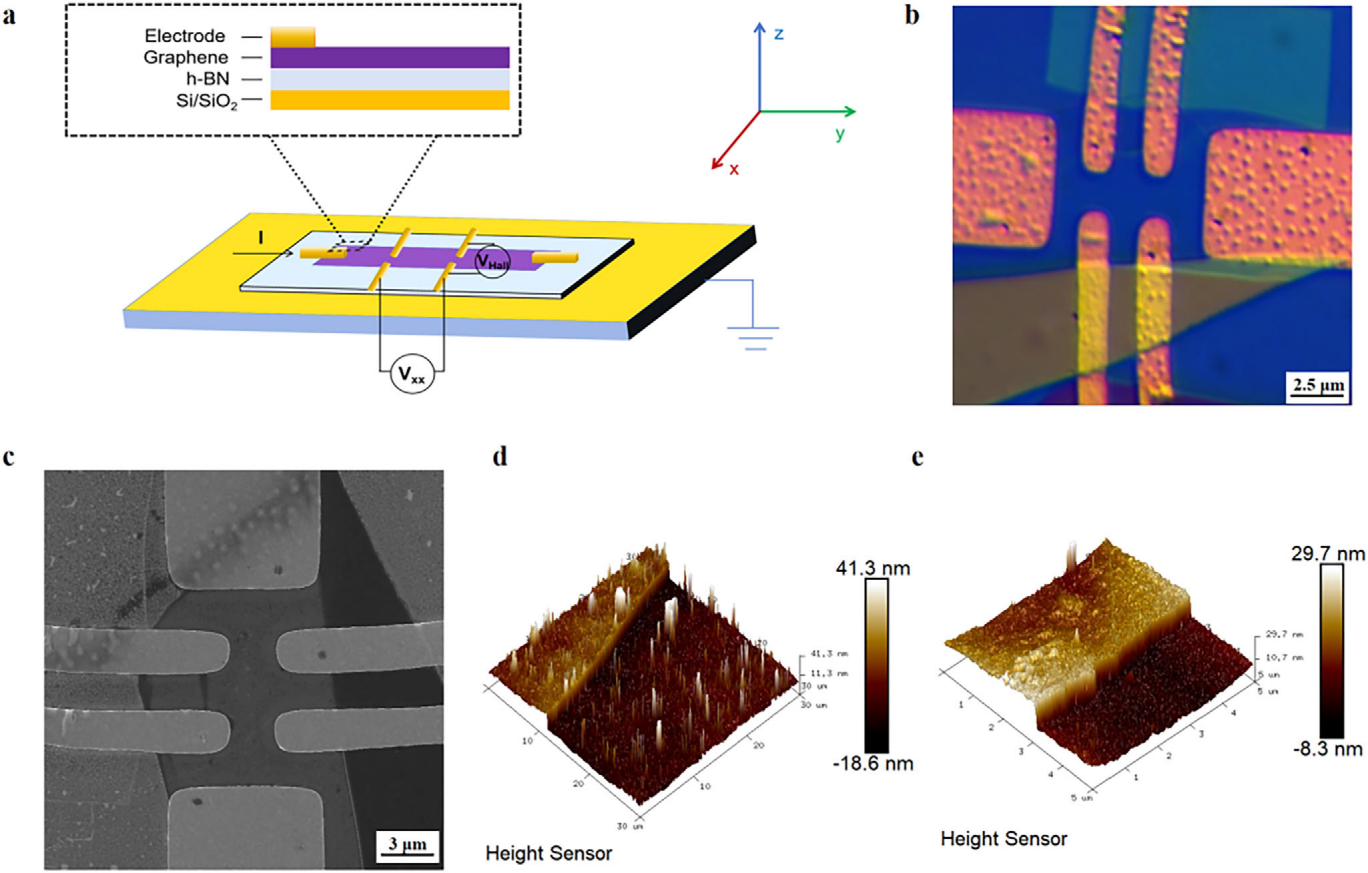
(a) shows a schematic image of the device structure. (b) shows an optical micrograph of 6‐µm‐long Hall cross (11 nm h‐BN). (c) shows SEM images of the 11 nm h‐BN device. (d) shows the AFM image of the 11 nm h‐BN device. (e) shows the AFM image of the 22 nm h‐BN device.

To have a direct visualization of the interface free of interfacial imperfections, we characterized every buried and exposed van‐der‐Waals interface via optical microscopy and Scanning Electron Microscopy (SEM). Figure 1a shows the schematic structure of the devices. The microscopy images of our van‐der‐Waals Hall bars reveal defect‐free heterojunctions whose interfacial quality is limited only by the atomic flatness of the constituent crystals. Optical micrographs display clean surfaces across the 6‐µm‐long channel in the first device, where an 11‐nm‐thick h‐BN crystal underlies a continuous monolayer graphene sheet that is subsequently overlaid by a transferred ferromagnetic electrode (Figure 1b). The SEM image (Figure 1c) shows atomically sharp, bubble‐free interfaces among all three layers. Figure 1d,e shows the graphs from atomic‐force microscopy (AFM). The images record a root‐mean‐square roughness below 0.3 nm across the entire h‐BN interface, of which the thickness is 11 nm (Figure 1d) and 22 nm (Figure 1e). These results confirm the absence of wrinkles, residues, or interfacial voids. These images of interfacial perfections demonstrate that the full vdW fabrication strategy is able to eliminate the extrinsic disorder, irrespective of h‐BN thickness. The elimination of extrinsic disorder within both stacks establishes an ideal platform for probing intrinsic spin‐transport phenomena and for realizing scalable, back‐end‐compatible spintronic circuits.

**FIGURE 2:**
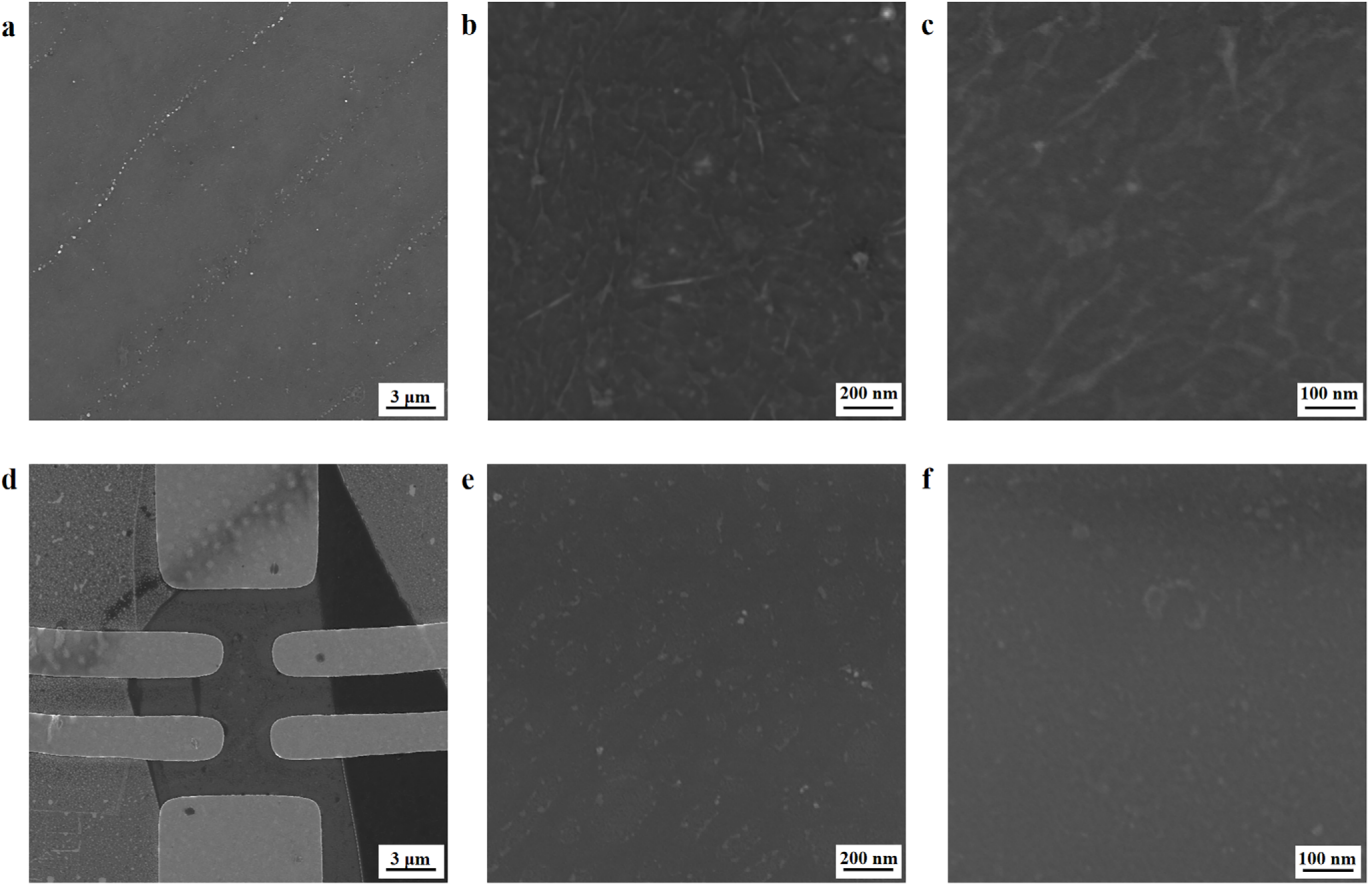
SEM images of CVD (a–c) and full vdW (d–f) fabricated devices.

To benchmark the interfacial perfection achieved by the full vdW fabrication strategy, we compare the interfacial conditions of our devices with those fabricated by common chemical vapor deposition (CVD) technology. We deliberately fabricated two archetypal graphene/h‐BN stacks in parallel: monolayer graphene synthesized by chemical vapor deposition (CVD) and subsequently transferred onto CVD h‐BN, and our van‐der‐Waals Hall bar. Both devices are fabricated with 11 nm h‐BN. This side‐by‐side design isolates the influence of interfacial imperfections related to the different fabrication technology, as both devices share the same encapsulation scheme, electrode geometry, and post‐fabrication thermal budget. The first three panels (Figure 2a–c) show the SEM images of a CVD‐grown graphene/h‐BN stack. In Figure 2a, there are white dots on the polycrystalline graphene sheet surface. These dots are interfacial imperfections, indicating the existence of nanometer‐scale corrugations and discrete wrinkle networks that propagate across grain boundaries. SEM images at higher magnifications (Figure 2b,c) show dark and bright stripes on the surface, which indicate the existence of sub‐micron folds and interfacial voids at graphene–h‐BN domains, attributed to non‐uniform van‐der‐Waals adhesion. The bright spots in both images indicate residual catalyst nanoparticles embedded at the interface, introducing local strain gradients that are known to shorten spin coherence. In stark contrast, the mechanically‐exfoliated counterpart presents an atomically pristine interface (Figure 2d–f). The SEM images in Figure 2d exhibit clean surfaces across the 6‐µm Hall cross, with no discernible wrinkles or tears. SEM images at higher magnification in Figure 2e,f show no features observed in SEM images of CVD samples, which indicates sub‐ångström flatness and complete absence of interfacial voids or particulate contamination, consistent with a defect‐free van‐der‐Waals junction. These SEM images of the samples fabricated by both strategies reveal that the full vdW fabrication strategy is able to prevent the interfacial imperfections due to the CVD process, such as nanometer‐scale corrugations, wrinkle networks, and residual catalyst nanoparticles, which introduce spatially inhomogeneous strain that acts as efficient spin‐dephasing centers.

The perfect interface fabricated by the full vdW strategy shown in Figures 1 and 2 is supposed to provide a platform for stable visible quantum transport characteristics and maintain the intrinsic Hall transport characteristics of graphene. The reason is that interfacial perfection suppresses the random spin–orbit modulations during phase‐coherent quantum transport, which destroys quantum phase coherence. As a result, the spin lifetime can be restored, which is beneficial for scalable, back‐end‐of‐line spin logic applications. We therefore interrogate phase‐coherent quantum transport and Hall transport in full vdW strategy fabricated graphene/h‐BN Hall bars.

Magneto‐transport measurement at millitesla magnetic field resolution can characterize the phase‐coherent quantum transport and Hall transport with the relations between magneto‐resistance and the applied magnetic field. Here, we report low‐temperature (2 K–23 K) magneto‐transport measurements of monolayer graphene atop h‐BN under perpendicular (B_z_) and in‐plane (B_xy_) magnetic fields, comparing two bias currents (0.5 and 10 µA).

To study the phase‐coherent quantum transport, we characterized the relations between magneto‐resistance (R_xx_ and R_xy_) and magnetic field (B_z_ and B_xy_) in the 11 nm‐h‐BN device. In Figure 3a we measured the R_xx_ vs. B_z_ relation with 10 µA current applied under a temperature ranging from 2 to 23 K with no in‐plane magnetic field applied. The relationship between R_xx_ and B_z_ shows non‐monotonic trends symmetric about B_z_ = 0 T position, which forms Landau fans (the inset shows that Landau fans maintain at the temperature ranging from 17 to 23 K). In the figure, we can see that under temperatures from 2 to 14 K, the R_xx_ vs. B_z_ relation remains stable with almost the same minimum at B_z_ = 0 T position. Under temperature from 17 to 23 K, the R_xx_ vs. B_z_ relation follows another curve. The R_xx_ vs. B_z_ relation shows high temperature robustness, which implies high spin–valley degeneracy [11]. Such high spin–valley degeneracy remains under a 0.5 µA current measurement, as is shown in Figure 3e (the inset shows that Landau fans maintain at the temperature ranging from 17 to 23 K), which shows a similar R_xx_ vs. B_z_ relation to that in Figure 3a. The high spin–valley degeneracy results from a suppressed random spin–orbit modulations during phase‐coherent quantum transport.

FIGURE 3Magneto‐transport measurement results of the 11 nm h‐BN‐supported Hall bar recorded at 10 and 0.5 µA applied current (a) R_xx_ vs. B_z_ at 10 µA (full temperature range 2–23 K) (b) R_xx_ vs. B_xy_ at 10 µA (weak anti‐localization dips) (c) R_xy_ vs. B_xy_ at 10 µA (linear Hall response) (d) R_xy_ vs. B_z_ at 10 µA (Hall slope asymmetry) (e–h) Corresponding measurements at 0.5 µA (direct comparison).

**Figure:**
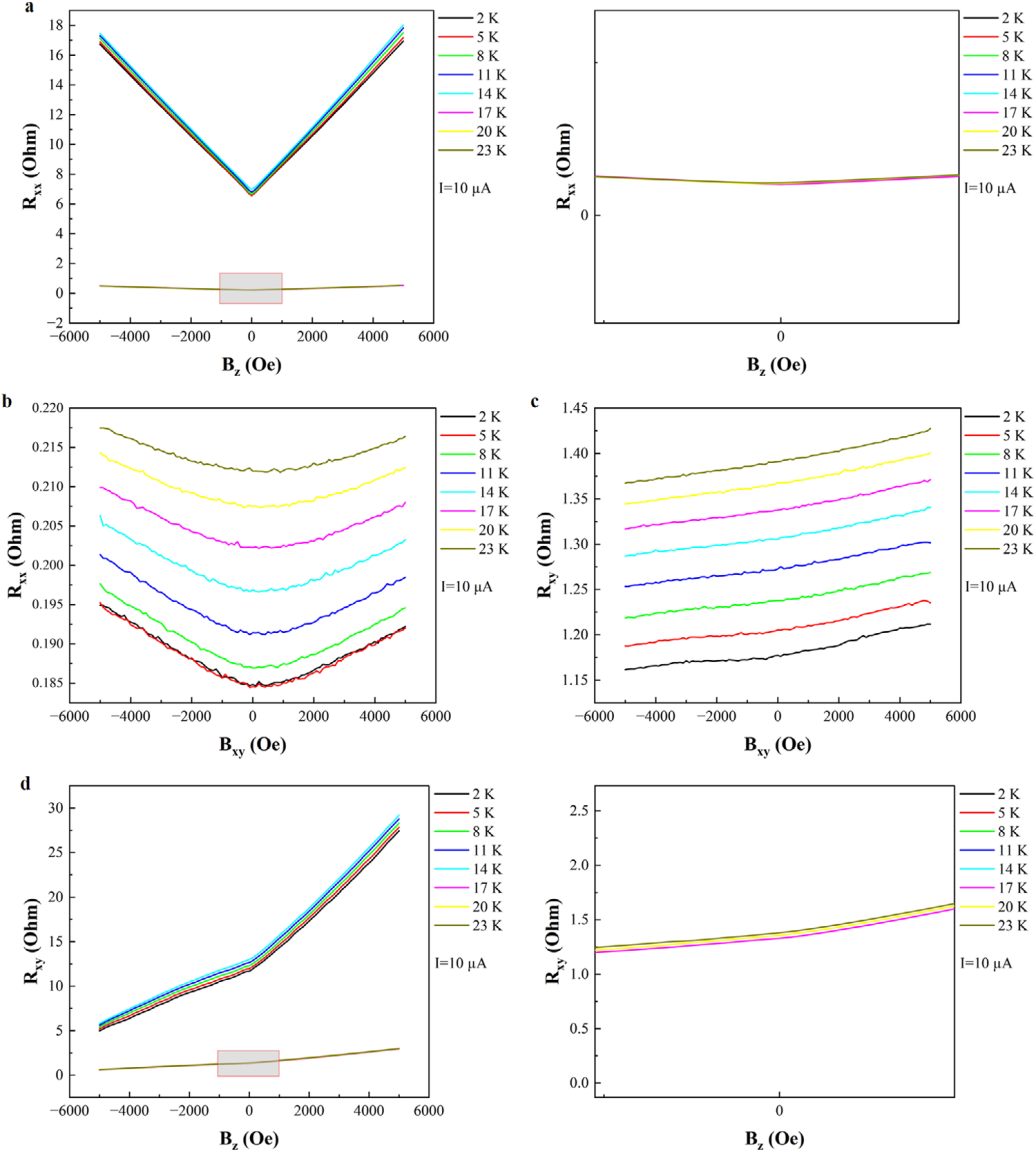

**Figure:**
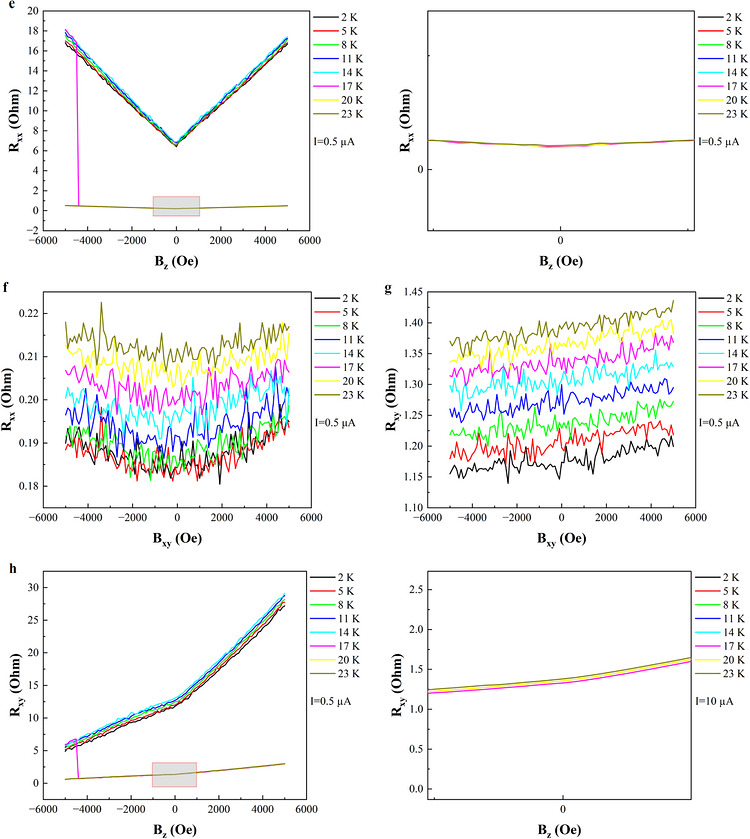

In Figure 3b,c, we measured the R_xx_ vs. B_xy_ and R_xy_ vs. B_xy_ relations, which show coupled visible quantum effects of intrinsic transport in the graphene channel. The curves in Figure 3b show the R_xx_ vs. B_xy_ relation with 10 µA current applied under a temperature range from 2 to 23 K with no in‐plane magnetic field applied. The relationship between R_xx_ and B_xy_ shows V‐shaped symmetric curves centered at B_xy_ = 0 T position. The depth of the dip at B_xy_ = 0 T position decreases monotonically with increasing temperature. These characteristics in the curves are due to the following mechanisms. In our device, owing to the vdW fabrication strategy, the devices have an atomic‐level flat interface and charge‐neutral graphene channel, which enables an enhanced phase‐coherence length (l_φ_) that approaches the device width, leading to a prominent weak anti‐localization of massless Dirac fermions [12]. The enhanced l_φ_ enables quantum interference between time‐reversed, electron‐hole trajectories that encircle opposite valleys [13]. When in‐plane B_xy_ = 0 T, the interference is preserved; meanwhile, the back‐scattering is enhanced. Increasing the B_xy_ breaks the phase balance, washing out the interference and restoring the classical background, which lifts the resistance. The reduction of the depth of the dips with increasing temperature reflects the T^−1^ dependence of l_φ_, consistent with electron‐electron scattering in a disorder‐free, low‐density limit situation [14]. Such behaviors are also observed with a reduced current magnitude (0.5 µA), as is shown in Figure 3f. In a word, the interfacial perfection due to our full vdW fabrication strategy preserves the phase‐coherence length (l_φ_), which facilitates the stable observation of quantum effects in phase‐coherent quantum transport. In Figure 3c, we measured the R_xy_ vs. B_xy_ relation with 10 µA current applied under a temperature ranging from 2 to 23 K with no in‐plane magnetic field applied. The R_xy_ has a discrete linear relationship to B_xy_ separated by different temperatures with a temperature‐independent slope. The linear R_xy_ vs. B_xy_ in Figure 3c reflects ordinary Hall response in charge‐neutral graphene [15]: the interface fabricated by our vdW strategy preserves a constant, low carrier density, while the in‐plane field couples only to spin–valley texture without inducing orbital bending, so R_xy_ = B_xy_/(ne) remains linear with slope 1/ne and no Landau features, which is the characteristic of massless Dirac fermions in the classical Hall regime [4]. Also, similar behaviors are observed in the 0.5 µA current measurement, as shown in Figure 3g.

The simultaneous existence of the low‐field quantum behaviors in Figure 3b,c is rarely observed in the samples fabricated by other techniques. This is because conventional metallization or etching introduces chemical dopants, interfacial imperfections, and strong Fermi‐level pinning that shift the electrochemical potential far from the charge‐neutral Dirac point, locking the carrier density at high values and pushing the quantum limit to larger magnetic fields [4]. Such conventional processing also shortens the phase‐coherence length via enhanced intervalley scattering, suppressing the phase‐coherent back‐scattering cancellation between electron and hole edge states [4]. In contrast, our vdW fabrication strategy electrodes leave the graphene/hBN interface atomically intact. The Fermi level can therefore reside naturally at the Dirac point, enabling micron‐scale phase coherence and the formation of counter‐propagating electron–hole edge channels that already fit into the lowest Landau levels at millitesla fields, giving zero‐field resistance dips and pure ordinary Hall response, which are seldom resolved in conventional devices.

To study the Hall transport characteristics, in Figure 3d, we measured the relationship between the Hall resistance (R_xy_) and perpendicular magnetic field (B_z_) with 10 µA current applied under a temperature ranging from 2 to 23 K with no in‐plane magnetic field applied in the 11 nm‐h‐BN device. The R_xy_ vs. B_z_ curve shows a linear relationship with different slopes on the left‐ and right‐hand side of B_z_ = 0 T position (also valid at temperature ranging from 17 to 23 K as is shown in the inset), which is similar to the intrinsic Hall transport characteristics of graphene. The only difference between the curve in Figure 3d and the intrinsic transport curve is that the intrinsic Hall transport curve of graphene has a uniform slope. This is due to the interplay between a twist moiré superlattice and hBN‐induced Rashba spin–orbit coupling [16]. These Hall resistance behaviors are also observed in the 0.5 µA current measurement shown in Figure 3h (also valid at temperatures ranging from 17 to 23 K, as shown in the inset). The emergence of such low‐field behaviors in Figure 3d is enabled by our full vdW fabrication strategy. The pick‐up electrodes in this fabrication process introduce neither chemical dopants nor processing‐induced damage, eliminating Fermi‐level pinning at the metal–graphene interface [4]. Consequently, the carrier density remains ultra‐low, and electron–hole asymmetry is pronounced, rendering the Hall coefficient highly sensitive to millitesla‐scale fields and producing sign reversals and hysteresis that are inaccessible in conventional metallized devices.

The results in Figure 3 show that the interfacial perfection originated from the vdW fabrication strategy can (1) suppress random spin–orbit modulations, (2) enhance phase‐coherence length (l_φ_), (3) preserve a constant, low carrier density, and (4) prevent chemical dopants and processing‐induced damage, which provides an methodology for fabricating ideal devices with more prominent spin‐related quantum effects and more stable intrinsic transport characteristics.

Interfacial perfection enables direct modulation of spin transport characteristics by changing the strength of the dielectric shielding effect without destructing any interface. The strength of the dielectric shielding can be modulated by changing the h‐BN thickness. To systematically probe the dielectric shielding limit on phase‐coherent quantum transport and Hall transport, we do the identical magneto‐transport measurements in a 22 nm‐hBN device, showing the different spin transport characteristics due to the change in h‐BN thickness.

**FIGURE 4:**
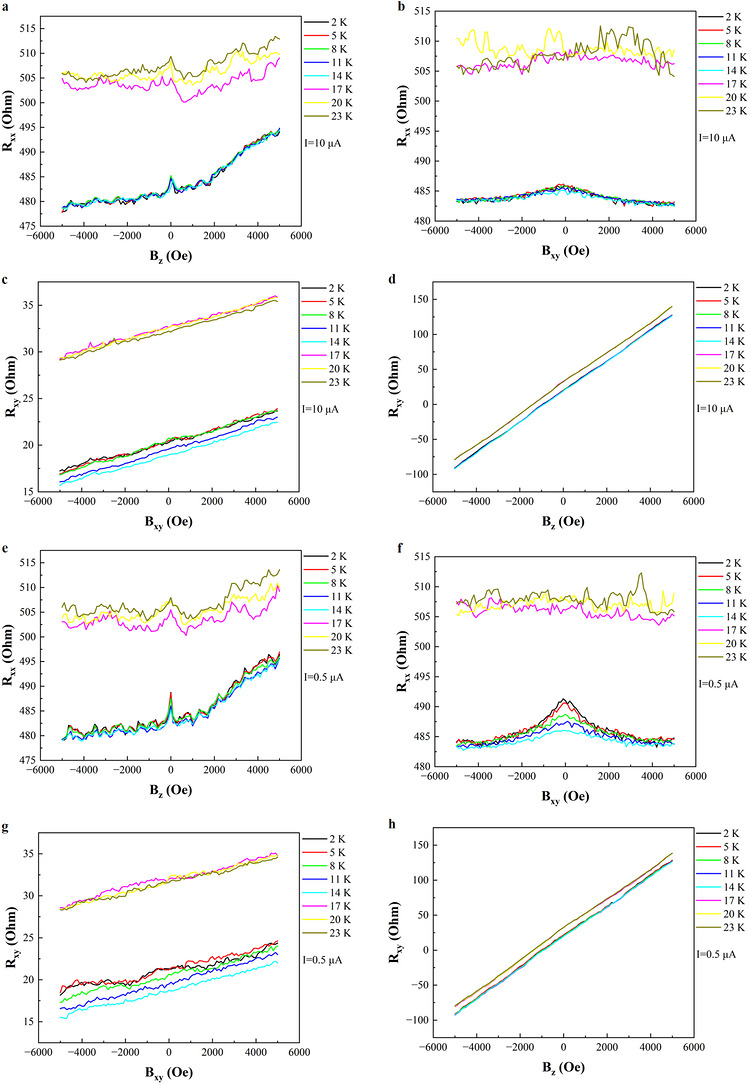
(Magneto‐transport measurement results of the 22 nm h‐BN‐supported Hall bar recorded at 10 and 0.5 µA applied current (a) R_xx_ vs. B_z_ at 10 µA (weak localization peak, no Landau fans) (b) R_xx_ vs. B_xy_ at 10 µA (weak localization peak) (c) R_xy_ vs. B_xy_ at 10 µA (temperature‐collapsed lines) (d) R_xy_ vs. B_z_ at 10 µA (strictly linear, zero‐offset) (e–h) Corresponding measurements at 0.5 µA.).

To elucidate phase‐coherent quantum transport, we systematically measure the relationship between magneto‐resistance (R_xx_ and R_xy_) and out‐of‐plane (B_z_) and in‐plane (B_xy_) magnetic fields in a 22 nm h‐BN device. Figure 4a presents R_xx_ versus B_z_ obtained with a 10 µA applied current at temperatures ranging from 2 to 23 K in the absence of an in‐plane field.

Figure 4a exhibits increasing R_xx_ with the increased B_z_, with a peak at B_z_  =  0 T whose height decreases with increasing temperature. The increasing relation between R_xx_ and B_z_ is due to a commonly observed classical scattering mechanism. However, the peak observed at B_z_  =  0 T is due to a quantum effect. The interfacial perfection provided by our full vdW strategy offers a atomical‐flat, low defect density, low doping level, and enhanced phase‐coherence length. Under such conditions, the 22‐nm hBN substrate weakens the back‐gate electric field, depressing the carrier density and placing the Fermi energy at the quantum‐limit edge. As a result, at zero field, phase‐coherent, spin–valley‐locked electron–hole trajectories interfere destructively, giving rise to a weak‐localization peak observed in the measurement [17]. However, in Figure 4a, we don't observe any Landau fan existing in Figure 3a. The absence of Landau fans is due to the excessive dielectric shielding of the thicker hBN layer (22 nm in Figure 4a compared to 11 nm in Figure 3a): under identical conditions, the thicker h‐BN layer reduces the electric field at the graphene surface by half, pinning the carrier density to the “ultra‐low‐doping” regime and low Fermi level (E_F_). Within such “ultra‐low‐doping” regime, even at B_z_ = 1 T, the Landau‐level spacing ℏω_c_ ≈ 1.3√B_z_ meV [18] is comparable to or larger than E_F_, so the system remains at the quantum‐limit edge. Consequently, no Landau fan exists. The observed R_xx_ vs. B_z_ curves with a peak at B_z_  =  0 T mirror the ultra‐low doping, long phase‐coherence inherent to the pristine van‐der‐Waals interface, providing the condition to modulate the phase‐coherent quantum transport via changing the thickness of h‐BN. In Figure 4e, a similar R_xx_ vs. B_z_ relation with a more prominent peak at B_z_ = 0 T is observed at 0.5 µA applied current. Reducing the current from 10 to 0.5 µA merely suppresses Joule‐heating‐induced dephasing, thereby amplifying the peaks at B_z_ = 0 T [4, 5, 19].

The curves in Figure 4b show the R_xx_ vs. B_xy_ relation with 10 µA current applied under a temperature ranging from 2 to 23 K with no in‐plane magnetic field applied. In Figure 4b we observed a symmetric, broad resistance peak centered at B_xy_  =  0 T position, which does not change with temperature. This feature is the signature of weak localization. The vdW fabrication strategy yields an atomically clean graphene/hBN interface, suppressing chemical doping and intervalley scattering so that the phase‐coherence length exceeds the device width, suppressing back‐scattering and producing the resistance maximum at 22 nm h‐BN. However, in Figure 3b, the R_xx_ value reaches the minimum at B_xy_  =  0 T, while in Figure 4b, there exists a maximum of R_xx_ at B_xy_  =  0 T. The change from a resistance minimum in the 11 nm h‐BN device to a pronounced maximum in the 22 nm h‐BN device (Figures 3b, 4b) is due to the enhanced dielectric shielding of the thicker h‐BN: the 22 nm h‐BN halves the surface electric field, reducing the carrier density to a very low level and pushing the Fermi level to the quantum‐limit edge. At this ultra‐low doping, phase‐coherent, spin–valley‐locked electron–hole trajectories interfere destructively at B_xy_ = 0 T, suppressing back‐scattering and producing a weak‐localization resistance peak. In the 11‐nm case, with approximately two times higher carrier density, it instead exhibits a weak‐anti‐localization minimum because the larger Fermi sea reduces the interference loop and favors sign‐reversed suppression [19]. Thus, the “minimum‐to‐maximum” switch in R_xx_ value maps directly onto the crossing from weak‐anti‐localization to weak‐localization controlled by h‐BN thickness, which is achieved without damaging the interfacial perfection. In Figure 4f, the broad resistance peak remains, which collapses monotonically when the temperature is raised from 2 to 23 K. However, in Figure 4b, the height of the peak remains almost unchanged with the increasing temperature. The reason for this difference is that reducing the current from 10 to 0.5 µA suppresses electron–electron scattering and Joule heating, elongating l_φ_ and thereby enhancing the peak. The continuous height reduction with temperature follows the standard T^−1^ dependence of the phase‐breaking rate in a disorder‐free, low‐density two‐dimensional system.

Figure 4c shows a linear R_xy_ vs. B_xy_ relation with 10 µA current applied under temperature ranging from 2 to 23 K current applied under temperature ranging from 2 to 23 K. The slope of the lines is almost temperature‐independent, and the lines are highly overlapped in the 2 to 14 K temperature range. These are hallmarks of ordinary intrinsic Hall transport under the in‐plane magnetic field, which is because 22 nm h‐BN pins the carrier density at a very low level, while B_xy_ couples solely to the spin–valley texture without altering transverse velocity. Meanwhile, the vdW interface offers a constant n value. As a result, R_xy_ = B_xy_/(ne) shows a linear relationship. Such a linear relationship of R_xy_ versus B_xy_ is also observed in Figure 4g when the applied current reduces to 0.5 µA. The intrinsic Hall transport characteristics under the in‐plane geometry are also observed in Figure 3c. However, the R_xy_ vs. B_xy_ lines shown in Figure 3c (11 nm h‐BN) have pronounced temperature‐dependent splitting characteristics. This difference originates from electro‐thermal environment differences due to differences in h‐BN thickness. Under the identical measurement conditions, weaker dielectric shielding in the 11 nm h‐BN produces a higher carrier density and stronger Joule heating, forcing the electron temperature to track the lattice temperature and causing a visible drift in the lines. The 22 nm h‐BN suppresses this thermal broadening via ultra‐low carrier density and enhanced heat dissipation, collapsing the curves into a single line. This demonstrates that h‐BN thickness can act as a built‐in electro‐thermal knob that does not damage the interface perfection [4].

The Hall transport characteristics measurement in 22 nm h‐BN device is also conducted in Figure 4d, with 10 µA current applied under a temperature ranging from 2 to 23 K with no in‐plane magnetic field applied. Figure 4d shows the relation of R_xy_ and B_z_: strictly linear with zero‐offset traces and no plateau. The slopes of the lines are temperature‐independent, which is the hallmark of ordinary intrinsic Hall transport characteristics under the out‐of‐plane magnetic field. Such R_xy_ vs. B_z_ relation is due to the strong dielectric shielding effect of 22 nm h‐BN where the interfacial perfection is preserved by the vdW fabrication strategy. During the measurement, vertical B_z_ supplies only a Lorentz force with ℏω_c_ ≳ E_F_, so R_xy_ = B_z_/(ne) stays linear, and no integer quantum‐Hall plateau develops [4, 19, 20]. The linear intrinsic Hall transport characteristics in the 22 nm h‐BN device are quite different from those in 11 nm h‐BN device shown in Figure 3d. The 11 nm h‐BN Hall bar exhibits different slopes around B_z_ = 0 T because of its higher carrier density and stronger electron–phonon coupling amplify Joule heating, driving a drift of carrier density n in R_xy_ = B_z_/(ne) [4, 20]. The 22 nm h‐BN suppresses this effect by halving the surface field, reducing the carrier density, and dissipating heat more efficiently. For this reason, the Hall slope remains strictly linear—demonstrating that h‐BN thickness can act as a built‐in, interface‐preserving electro‐thermal knob for Hall transport modulation [4, 20]. The same R_xy_ vs. B_z_ relation in Figure 4d is also observed in Figure 4h, where the applied current is reduced to 0.5 µA.

The results in Figure 4 show that the full vdW fabrication strategy delivers atomically flat, bubble‐free interfaces regardless of h‐BN thickness, which has the following advantages. (1) Extends l_φ_ to the micron scale, allowing weak‐localization and linear Hall response to be observed at millitesla fields and up to 23 K. (2) Suppresses charged‐impurity and strain‐induced scattering, so carrier density can be electrostatically pinned at very low levels without Fermi‐level drift. (3) Enables thickness‐controlled change from weak‐anti‐localization (11 nm) to weak‐localization (22 nm) without any process‐induced degradation, demonstrating a non‐invasive electro‐thermal knob for phase‐coherent quantum spin transport. These advantages enable the decoupling of spin coherence from h‐BN thickness and establish full‐vdW graphene/hBN as a scalable, back‐end‐of‐line platform for ultra‐low‐dissipation spin logic and topological superconducting circuits.

In Figures 3 and 4, we have observed that increasing the h‐BN thickness merely extinguishes the Landau fan and toggles the quantum‐interference characteristics between weak‐anti‐localization and weak‐localization (Figures 3a and 4a), while the temperature‐dependent coherence envelopes (Figures 3b and 4b) and the intrinsic Hall transport characteristics remain superimposable (Figures 3c, 4c, 3d, and 4d). These changes are due to the dielectric shielding effect in the magneto‐transport processes in devices with different h‐BN thickness. To elucidate the microscopic origin of this thickness‐dependent magneto‐transport observed in our full‐van‐der‐Waals devices, we carried out density‐functional theory (DFT) simulations of graphene/hBN stacks.

**FIGURE 5:**
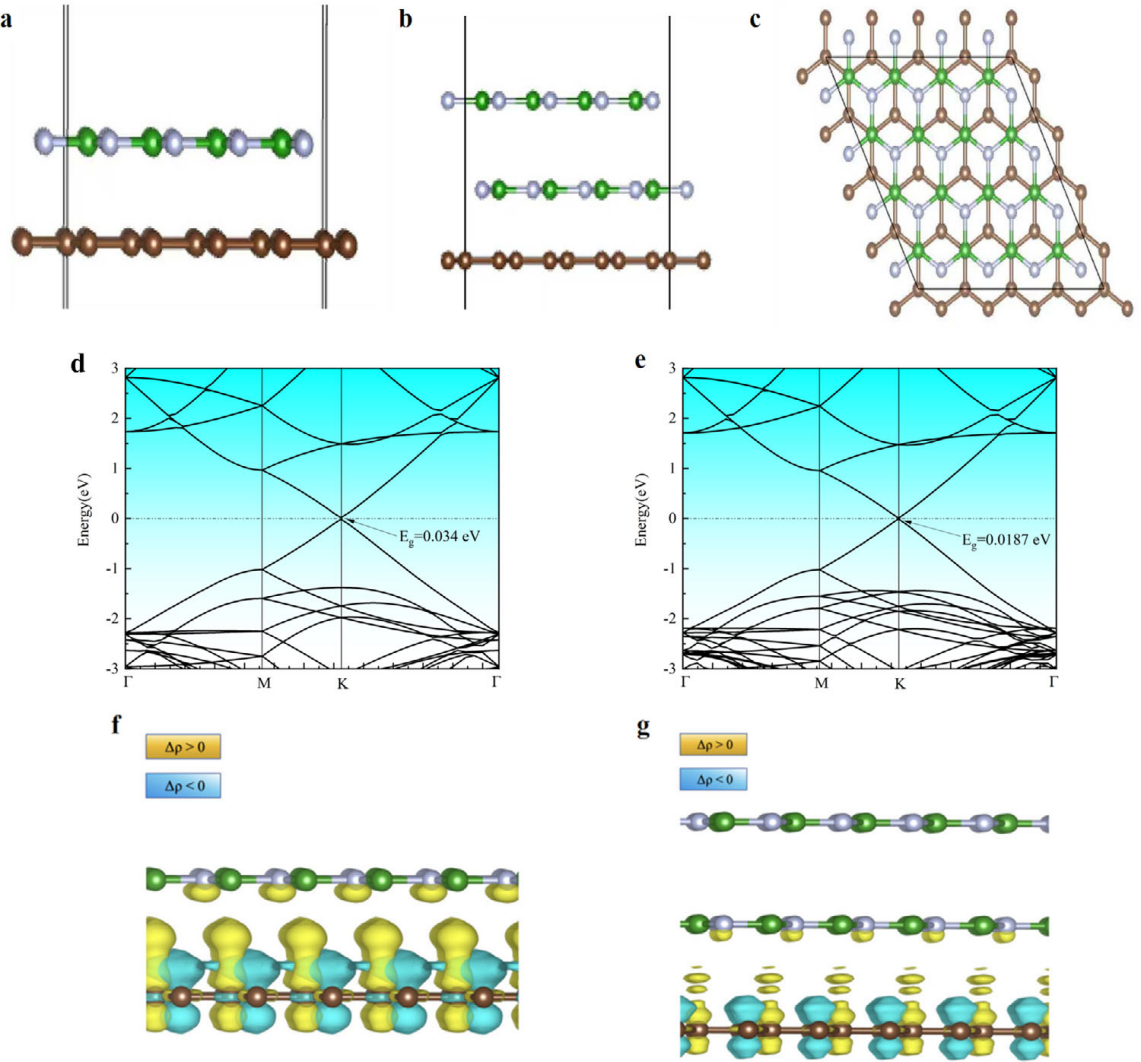
Model construction and DFT simulation results (dielectric response only, SOC excluded as experimentally saturated boundary condition): (a) Side‐view atomic structure of graphene on monolayer h‐BN (b) Side‐view atomic structure of graphene on bilayer h‐BN (c) Top‐view of the graphene/h‐BN van‐der‐Waals stack (d) Electronic band structure of graphene on monolayer h‐BN (e) Electronic band structure of graphene on bilayer h‐BN (f) Interfacial charge‐density difference for monolayer‐h‐BN‐supported graphene (g) Interfacial charge‐density difference for bilayer‐h‐BN‐supported graphene (Differential charge density (Δρ) for monolayer (f) and bilayer (h‐BN)/graphene (g) overlaid on the atomic structure. Yellow (Δρ > 0) and blue (Δρ < 0) isosurfaces (±0.0001 e Å^−^^3^) denote electron accumulation and depletion, respectively.).

Specifically, we performed DFT simulations comparing a monolayer h‐BN substrate (representative of the 11 nm h‐BN device) with a bilayer h‐BN slab (22 nm h‐BN device). The relaxed atomic stacks (Figure 5a,b) reproduce the experimentally reported defect‐free, bubble‐free morphology. The top‐view projection (Figure 5c) confirms the absence of rotational disorder, mirroring the small twist angle inferred from moiré‐period analysis.

Figure 5a,b shows the side‐view atomic models of graphene on monolayer (11 nm‐equivalent) and bilayer (22 nm‐equivalent) h‐BN, respectively, both exhibiting defect‐free, bubble‐free interfaces that mirror the experimental devices. Figure 5c displays the top view of the model which shows the negligible twist angle between graphene/h‐BN interfaces. These models are consistent with our devices fabricated by a full vdW strategy.

Figure 5d,e shows the calculated band structures of the monolayer model (11 nm‐equivalent) and bilayer model (22 nm‐equivalent), respectively. The results reveal that both stacks preserve the Dirac cone, but the monolayer substrate induces a 34 meV band gap, compared to the bilayer case (18 meV). The decrease of the band gap—from 34 meV for the monolayer model to 18 meV for the bilayer model—establishes that thicker h‐BN contributes solely to an enhanced dielectric shielding effect, weakening the surface Coulomb field of remote impurities and pushing the Fermi level closer to the charge‐neutral Dirac point while preserving the strictly almost gapless, linear dispersion of graphene [2].

Figure 5f,g shows the calculated charge‐density difference (Bader) diagrams of the monolayer model (11 nm‐equivalent) and the bilayer model (22 nm‐equivalent), respectively. In the figures, yellow (Δρ > 0) and blue (Δρ < 0) isosurfaces ( ± 0.0001 e Å^−^^3^) denote electron accumulation and depletion, respectively. Bader's analysis of the charge‐density difference shows almost identical electron accumulation on the C atoms (≈0.20 e^−^ per C) and depletion on N atoms (≈–2.2 e^−^ per N) in both models, yielding a net 0.010–0.013 e^−^ transfer from h‐BN to graphene. The almost identical interfacial charge‐density difference reveals that charge redistribution is confined to the first h‐BN layer adjacent to graphene [21], additional dielectric thickness neither alters the interfacial dipole nor introduces extra charge transfer—implying that the spin–orbit coupling is already saturated and that thicker h‐BN serves solely as an electrostatic shielding, leaving the interfacial perfection and spin transport mechanisms undisturbed.

In summary, in Figure 5, we build models of defect‐free, bubble‐free graphene/h‐BN interfaces with negligible twist angle between layers via structural relaxation and conduct DFT simulations of both graphene/hBN models. These results reveal that thicker h‐BN solely enhances the dielectric shielding effect. Due to the stronger dielectric shielding effect, with increasing h‐BN thickness, the bands shift rigidly downward. pushing the Fermi level toward the charge‐neutral Dirac point while leaving the Dirac cone intact. This purely electrostatic motion shrinks the Fermi wave factor (k_F_), converting weak anti‐localization into weak localization, raises ℏω_c_ above the Fermi energy to extinguish the Landau fan by energy‐scale mismatch rather than disorder (consistent with the result in Figures 3a and 4a), without altering the T^−^^1^ coherence envelope (consistent with the result in Figures 3b and 4b). Meanwhile, the thicker h‐BN drops the carrier density into the ultra‐low‐doping regime where thick h‐BN screens Joule‐heating‐induced n‐drift, collapsing the Hall traces into a single, temperature‐insensitive line (consistent with the result in Figures 3c, 4c, 3d, and 4d). In other words, our simulation results support the experimental measurements, meanwhile explaining the spin transport mechanisms on a microscopic scale.

**FIGURE 6:**
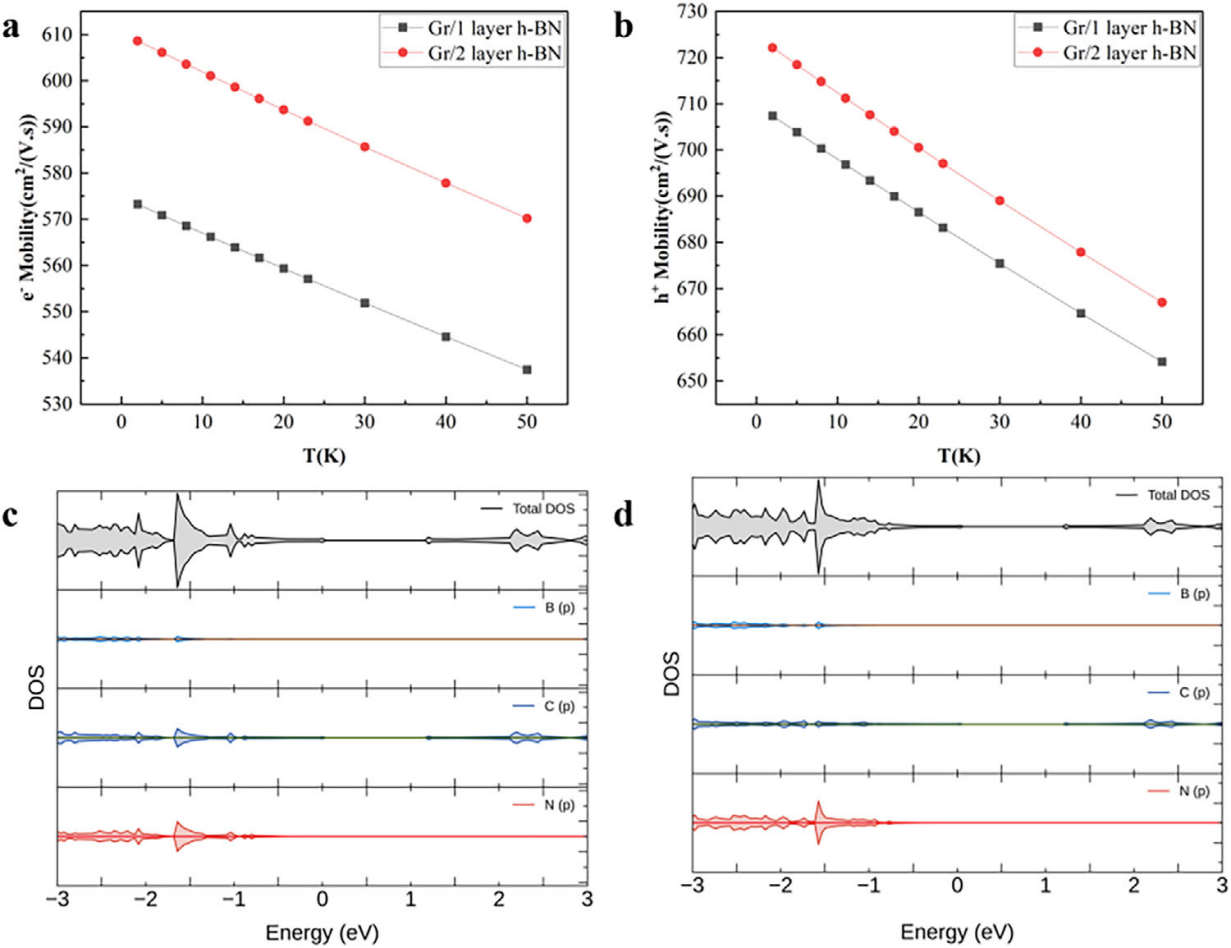
DFT‐calculated carrier mobility vs. temperature. and density of states (DOS) (a) Electron mobility for graphene on monolayer h‐BN (black squares) and bilayer h‐BN (red circles) (b) hole mobility for graphene on monolayer h‐BN (black squares) and bilayer h‐BN (red circles). Bilayer h‐BN shows consistently higher mobility (∼5%–6% enhancement) due to stronger dielectric screening of remote impurities, while identical temperature dependence confirms unchanged scattering mechanisms. Total DOS (shaded gray) and orbital‐projected DOS for graphene on (c) monolayer h‐BN and (d) bilayer h‐BN. Near‐identical profiles, particularly near the Dirac point, confirm that interfacial electronic structure and charge transfer saturate at the first h‐BN layer. The absence of additional states in the bilayer system validates that extra thickness contributes purely to dielectric screening without altering interfacial chemistry.

To quantitatively connect DFT predictions with transport observations, we performed mobility calculations and density of states (DOS) analysis for graphene on monolayer and bilayer h‐BN (Figure 6).

Mobility calculations (Figure 6a,b) reveal systematically higher values for bilayer h‐BN (electron: 609 vs. 573 cm^2^/V·s; hole: 722 vs. 707 cm^2^/V·s at 2 K), consistent with enhanced dielectric screening reducing remote impurity scattering. The identical temperature dependence (μ ∝ T(− α),  α  ≈  0.3) confirms that scattering mechanisms remain unchanged—phonon and electron‐electron scattering dominate in both cases, with no evidence of thickness‐induced disorder.

The DOS for both systems (Figure 6c,d) show nearly identical profiles near the Dirac point, with coinciding C, N, and B p‐orbital projected densities. This confirms that interfacial electronic structure saturates at the first h‐BN layer: no additional states emerge in the bilayer system, and charge redistribution remains localized at the interface. This validates our treatment of SOC and interfacial chemistry as thickness‐independent boundary conditions.

These simulations complete the causal chain: DFT‐predicted dielectric enhancement—experimentally observed carrier density modulation—thickness‐tuned quantum coherence, all without invoking SOC or interfacial chemistry variations.

## 3 Conclusion

Graphene/h‐BN devices show ideal properties that are beneficial for scalable two‐dimensional logic and quantum technologies [4]. Current approaches to fabricating the graphene/h‐BN devices remain entangled by interfacial imperfection and inseparable thickness‐scattering correlations, preventing h‐BN thickness from serving as a non‐invasive electrostatic dial. In this work, we fabricate graphene/h‐BN Hall bar devices using full vdW fabrication, achieving interface perfection in 11‐ and 22‐nm h‐BN devices strategically selected to bracket the continuum dielectric regime (Figures 1 and 2). These devices exhibit switchable quantum interference from weak anti‐localization to weak localization with increasing h‐BN thickness, on–off Landau fans (Figures 3a, 4a, 3b, and 4b) and temperature‐collapsed Hall slopes (Figures 3c, 4c, 3d, and 4d) solely because dielectric shielding differs, while the coherence temperature dependence and proximity spin–orbit magnitude remain overlapped. DFT calculation results show that the interfacial effect saturates at the first h‐BN layer; extra h‐BN thickness merely suppresses residual doping without adding defects (Figure 5). Thus, changing h‐BN thickness means changing doping level, decoupling spin transport characteristics from processing strategies, and making spin transport characteristics determined by intrinsic electron–electron interactions. Thereby delivering a scalable, back‐end‐compatible platform for two‐dimensional spin logic and topological superconducting circuits with pristine interfaces. Theoretically extrapolated boundaries (5–30 nm) are grounded in DFT‐calibrated effective medium models, with 11 and 22 nm serving as experimental anchor points for partial and full screening saturation, respectively.

In summary, we demonstrate a contamination‐free, full van‐der‐Waals fabrication strategy that decouples the dielectric shielding effect due to h‐BN thickness and interfacial imperfection, which previously results from the h‐BN fabrication via other processes in spin transport modulation in graphene/h‐BN devices, demonstrating that h‐BN thickness is capable of tuning carrier density while leaving spin coherence intact—thereby delivering a scalable, back‐end‐compatible pathway for two‐dimensional spin logic with atomically pristine interfaces [4, 20]. Our conclusions regarding thickness‐tuned dielectric shielding apply within the continuum regime (5–30 nm) grounded in theory and validated at 11 and 22 nm. Below 5 nm, discrete layer‐number effects may introduce deviations; above 30 nm, practical gate efficiency limits applicability. The causal chain “flatness– reduced scattering—enhanced coherence” is validated through direct internal comparison of four devices (two perfect vs. two imperfect) rather than deliberate defect engineering, which we argue offers greater ecological validity for assessing technological scalability.

## 4 Method

### 4.1 Mechanical Exfoliation and Van‐der‐Waals Assembly

Single‐crystal h‐BN (HQ Graphene) [22] and graphite were cleaved on Nitto SPV‐224 dicing tape and transferred onto 285 nm SiO_2_/p++ Si (0.001 Ω cm, Nova Electronic Materials). Mono‐ and few‐layer regions were identified by optical contrast (ΔRGB ≈ 12–15 for monolayer h‐BN) [23]. Graphene flakes were prepared identically. A 5 wt.% aqueous polyvinyl‐alcohol (PVA, Mw 13–23 k) film was spin‐coated (1000 r.p.m., 45 s) onto a 1 mm PDMS stamp, dried at 80°C for 5 min, and mounted on a home‐built micromanipulator (±0.5 µm lateral, 50 nm vertical resolution). Graphene was picked up at room temperature with an estimated contact force of ∼2 N m^−^^1^ [24], aligned to a pre‐selected h‐BN flake (<1 µm accuracy), and lifted off to complete the graphene/h‐BN stack. Au(50 nm)/Ti(5 nm) Hall‐bar electrodes prefabricated on a PDMS/glass carrier were transferred with a four‐axis microplotter [4]. The electrode array was aligned to the stack at 25°C; the substrate was then heated to 150°C (10°C min^−^^1^) for 5–10 min to activate the PVA adhesive. The glass carrier was retracted at 50 µm s^−^^1^, leaving the electrodes on the heterostructure. PVA was dissolved in warm acetone (50°C, 5 min) or, for encapsulation, baked at 120°C for 10 min to yield a ∼20 nm passivation layer.

### 4.2 Spin Transport Measurements

Non‐local spin‐valve signals were recorded in a Quantum Design DynaCool system (9 T) at 2–14 K (± 0.01 K, Cernox sensor) [25]. A d.c. current (0.5 or 10 µA) was injected through the outer contacts while the out‐of‐plane field was swept from −0.5 to +0.5 T at 0.2 T min^−^^1^. The non‐local voltage was measured with a lock‐in amplifier (SR‐865, 13 Hz, 100 ms time constant) and averaged over three sweeps.

### 4.3 Computational Details

All the Spin‐polarized calculations were performed in the framework of the density functional theory with the projector augmented plane‐wave method, as implemented in the Vienna ab initio simulation package [26, 27]. The generalized gradient approximation proposed by Perdew–Burke–Ernzerhof (PBE) functional was adopted for the exchange‐correlation potential [28]. The long‐range van der Waals (vdW) interaction was described by the DFT‐D3 method [29]. The cut‐off energy for the plane wave is set to 400 eV. A vacuum layer with a thickness of 12 Å was added perpendicular to the sheet to avoid artificial interaction between periodic images. During geometry optimization, all atomic coordinates were relaxed with the convergence criteria of 0.03 eV·Å^−1^ for the forces on each atom. For each self‐consistent iteration, the total electronic energy was converged to 10^−5^ eV. The Brillouin zone integration is performed using a 3 × 3 × 1 k‐mesh to ensure computational accuracy.

The charge density difference was calculated as **Δ** ***ρ*** (***r***)  =  ***ρ*** ^*hBN*/*graphene*^  −  ***ρ** ^hBN^* −  ***ρ** ^graphene^*, where  ***ρ*** ^*hBN*/*graphene*^ is the electron density of the interacting system, while  ***ρ** ^hBN^* and  ***ρ** ^graphene^* are the electron densities of the hBN surface and graphene sheet calculated for the isolated systems with the atoms fixed in their positions as in the composite cell. The electron density difference isosurfaces were visualized with VESTA [30].

The local effective charge of each atom was calculated through the Bader analysis framework [31]. This is achieved through the zero‐flux condition, $\nabla \rho \left(r\right)\vec{n}\left(r\right)=0$. Subsequently, the total Bader charge of an atom, Q_Bader_, is calculated as the integration of the electron density on its delimited volume, and the effective charge is Q  =  ZVAL − Q_Bader_, where ZVAL is the valence charge.(Supporting Information)

## Conflicts of Interest

The authors declare no conflicts of interest.

## Supporting information




**Supporting File**: smtd70734‐sup‐0001‐SuppMat.docx.

## Data Availability

The Data that support the findings of this study are available from the corresponding author upon reasonable request.
