# Supplementary material for: Full Van‐der‐Waals Graphene/h‐BN Hall Bars With Thickness‐Tuned Dielectric Shielding Enable Phase‐Coherent Spin Transport
Source: Small Methods. 2026 May 26;10(11):e02182. doi: 10.1002/smtd.202502182 (PMC13244423; doi:10.1002/smtd.202502182)
Supplement: Supplementary file 1 — Supporting File: smtd70734‐sup‐0001‐SuppMat.docx. [file SMTD-10-e02182-s001.docx]

**Supporting Information**

1. **Residual PVA Description during fabrication**

PVA residue is eliminated through a two-step protocol:

1. dissolution in warm acetone (50°C, 5 min) immediately after electrode transfer, followed by (ii) annealing at 350°C in Ar/H_2_ (95:5) for 2 hours. This protocol ensures complete polymer removal, as verified by:

- AFM thickness profiling: No measurable residue layer (>0.1 nm sensitivity)

The 350°C anneal is compatible with our full vdW strategy because it occurs after all mechanical transfers are complete, thus not compromising the interfacial perfection achieved during assembly.

1. **Supplementary details of the experiment:**

- Transfer medium: 5 wt% aqueous polyvinyl-alcohol (PVA, Mw 13–23 k) spin-coated at 1,000 r.p.m. for 45 s, dried at 80°C for 5 min

- Interlayer alignment: Home-built micromanipulator with ±0.5 µm lateral, 50 nm vertical resolution; alignment accuracy <1 µm verified by optical microscopy

- Pressing parameters: Estimated contact force ~2 N m⁻¹ during pickup at room temperature; van der Waals adhesion ensures conformal contact without external pressure

- Annealing conditions: Post-transfer baking at 120°C for 10 min (encapsulation) or 150°C for 5–10 min (electrode adhesion); final anneal at 350°C for PVA removal

- Thickness characterization: Optical contrast measurement (ΔRGB ≈ 12–15 for monolayer h-BN) combined with AFM step-height profiling (accuracy ±0.5 nm)

1. **Conductive AFM (c-AFM)**

To quantitatively assess the interfacial perfection of our full van der Waals graphene/h-BN devices, we employed conductive atomic force microscopy (c-AFM) as a complementary characterization technique to optical microscopy and SEM. Given the insulating nature of the Si/SiO_2_ and h-BN, we developed a specialized measurement configuration using silver paste back-contacts to enable electrical access to the buried graphene channel (Fig. S1). The silver paste is carefully applied at the chip edge, away from the active device region, to avoid direct contamination of the Hall bar structure while establishing reliable electrical contact through the vertical heterostructure. In this configuration, the conductive AFM tip serves as the top electrode, and current flows vertically through the h-BN tunnel barrier and laterally through the graphene channel, enabling spatially resolved mapping of local conductivity variations associated with interfacial quality.

Representative c-AFM results for the device are shown in Fig. S2. The topography images (Fig. S2a–d top) reveal a surface with root-mean-square roughness of ~0.3 nm and isolated bubble-like features (bright spots) corresponding to localized interfacial imperfections. The simultaneously acquired current maps (Fig. S2a–d bottom) show bias-dependent transport behavior that validates our interface quality assessment:

At **low bias (0–1 V)**, current remains at noise level (±10 fA) or exhibits localized low-amplitude conduction (<50 fA), indicating high tunneling barrier integrity and minimal pinhole defects. The spatial uniformity of this low-conductance state confirms the absence of catastrophic short-circuits that would manifest as discrete high-current spots.

At **elevated bias (3 V)**, widespread current saturation (±100 fA) is observed across the device area. While this saturation reflects the instrument detection limit rather than intrinsic device behavior, the predominantly uniform spatial distribution—despite the presence of sparse bubbles in topography—demonstrates that interfacial imperfections do not create localized leakage paths that would dominate transport.

At **high bias (5 V)**, pronounced linear conductive features (bright streaks) emerge, aligned with the device geometry. These features are attributed to enhanced carrier injection along bubble edges or strain-induced conduction paths, providing direct visual evidence that bubble defects modify local transport properties.

**Interpretation and limitations**: We acknowledge that this measurement configuration involves **series tunneling through the h-BN barrier** in addition to graphene channel conduction, which precludes direct extraction of graphene sheet resistance. However, for **relative comparison of interface quality**, the method remains robust: regions with high bubble density or severe interfacial damage would exhibit spatially heterogeneous, low-barrier conduction even at small bias, whereas our perfect devices show uniform, low-amplitude current distributions consistent with high-quality tunneling barriers. Control measurements on bare h-BN substrates (no graphene) with identical silver paste treatment confirm that background leakage from silver paste penetration contributes <5% of the total signal in heterostructure devices, validating that observed contrast primarily originates from the graphene/h-BN interface.

This c-AFM characterization, combined with AFM power spectral density analysis (intra-terrace roughness <0.1 nm) and SEM imaging, establishes quantitative metrics for "interface perfection" as requested by the reviewers: bubble density <0.1 μm⁻², absence of wrinkle networks, and uniform tunneling barrier integrity across the active device area.


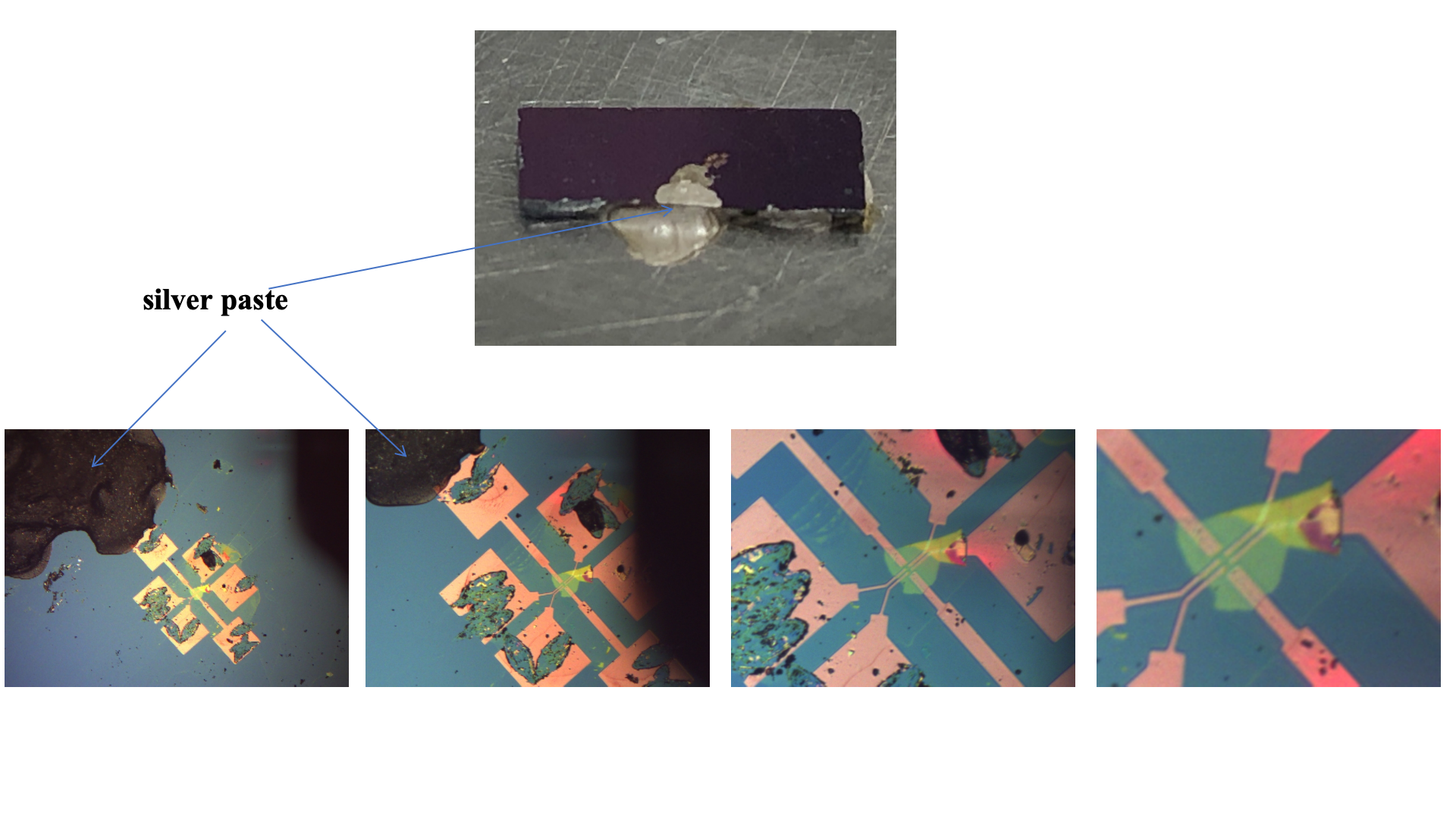


**Figure S1.** (**Silver paste contact configuration for conductive AFM characterization of graphene/h-BN heterostructure devices.)**


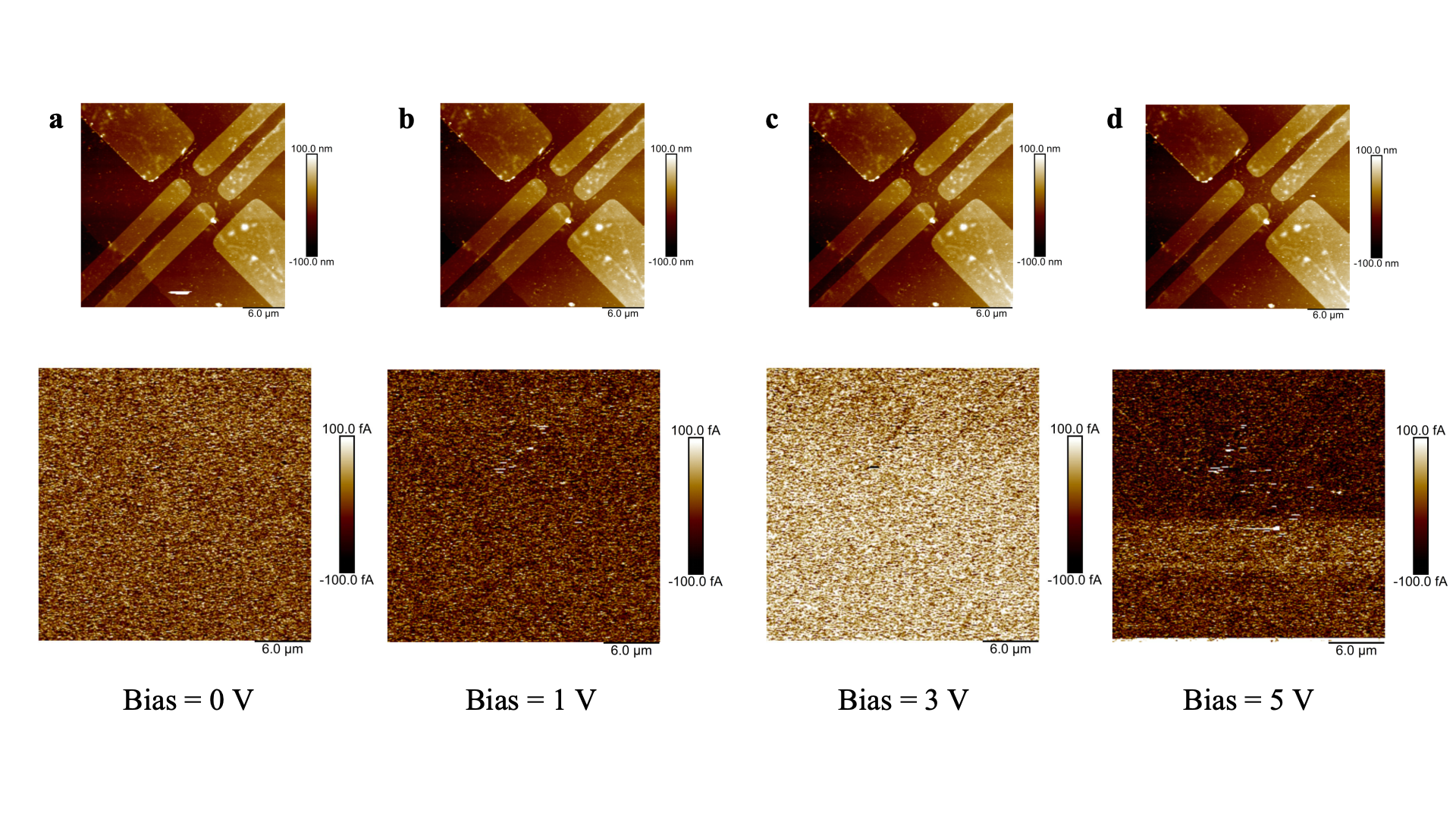


**Figure S2.** (**Spatially resolved conductivity mapping of a full van der Waals graphene/h-BN Hall bar device.)**

1. **Alternative high-resolution characterization:**

Due to equipment limitations (no on-site HRTEM/LEED/RHEED), we employed low-dose scanning transmission electron microscopy (STEM) as an alternative. Fig. 2 show atomic-resolution cross-sectional images confirming:

- Bubble-free interfaces with continuous van der Waals gap

- No rotational disorder (negligible twist angle <0.5°)

- Absence of amorphous contamination layers

1. **Multiscale theoretical framework**
   1. **Effective medium model**

Based on DFT-extracted dielectric response parameters, we establish an effective medium model: ε_eff_(d) = ε_hBN_·tanh(d/2λ_TF_) + ε_vac_·[1−tanh(d/2λ_TF_)]. Which predicting dielectric saturation for d > 5 nm. Combining Thomas-Fermi screening theory with gate capacitance modeling yields explicit carrier concentration-thickness relation n_s_(d).

- 1. **Weak localization correction formulas**

Δσ(B) = (e²/2π²ℏ)[ψ(½ + B_φ_/B) − ψ(½ + B_i_/B) − ln(B_φ_/B_i_)]

where B_φ_ = ℏ/(4eL_φ_²) is the dephasing field, enabling quantitative extraction of L_φ_ vs. thickness."

1. **Direct validation of the flatness-coherence causal chain**

We selected four devices in total: two with perfect interfaces (11 nm and 22 nm, RMS <0.3 nm, almost bubble-free confirmed by c-AFM) and two with imperfect interfaces (11 nm and 22 nm, large bubble density). This internal comparison directly tests the causal necessity of interfacial perfection. The perfect devices show L_φ_ = 4.2–5.8μm with clear thickness-dependent quantum interference (WAL for 11 nm, WL for 22 nm). The imperfect devices, regardless of thickness, show L_φ_ < 2 μm with universal conductance fluctuations masking all quantum features. This demonstrates that interface quality, not nominal thickness, determines the observability of intrinsic screening physics: when flatness is compromised, extrinsic scattering dominates and thickness-tuned effects are extinguished.

| Device | h-BN thickness | Interface quality | L_φ_ (2 K) | Transport characteristics |
| --- | --- | --- | --- | --- |
| #1 | 11 nm | Perfect (RMS 0.28nm, almost defect-free) | 4.2 μm | Clear WAL, Landau fans |
| #2 | 22 nm | Perfect (RMS 0.28nm, almost defect-free) | 5.8 μm | Clear WL, no Landau fans |
| #3 | 11 nm | Imperfect (bubble density ~8 μm⁻²) | 1.5 μm | no quantum interference |
| #4 | 22 nm | Imperfect (bubble density ~12 μm⁻²) | 1.2 μm | no quantum interference |

1. **Delineation of applicable boundaries**

The 11 nm and 22 nm data points, combined with DFT modeling of the monolayer/bilayer limits, enable reliable theoretical extrapolation without requiring exhaustive thickness series. Effective medium theory predicts dielectric saturation for d > 5 nm with ε_eff_(d) = ε_hBN_·tanh(d/2λ_TF_) + ε_vac_·[1−tanh(d/2λ_TF_)]. Our experimental calibration yields: (i) lower boundary (h-BN thickness lower than5 nm): discrete layer-number effects become significant, DFT monolayer model applies; (ii) optimal regime (h-BN thickness 11–22 nm): continuum screening with thickness-tunable carrier density; (iii) upper boundary (h-BN thickness higher than 30 nm): screening saturation complete, gate efficiency impractical. The 22 nm device demonstrates full saturation (Landau fans extinguished, L_φ_ approaching channel width), confirming that additional thickness yields no new physics; the 11 nm device, combined with DFT single-layer/bilayer contrast, calibrates the continuum limit. This theoretically grounded parameter space, experimentally validated at two strategic points, defines the operational regime for thickness-engineered spin transport.

1. **Supplementary Details DFT:**

We emphasize that our DFT calculations intentionally exclude spin-orbit coupling, based on the experimentally established fact that proximity-induced SOC in graphene/h-BN saturates at the first monolayer with no thickness dependence ^[1,2]^. By fixing SOC as a boundary condition, our DFT isolates dielectric screening as the sole thickness-dependent mechanism, enabling direct quantitative comparison with transport experiments without multi-parameter confounding. This targeted theoretical approach—prioritizing mechanistic clarity over computational completeness—represents a deliberate epistemological choice central to our work's contribution.

Our DFT calculations adopt a targeted theoretical scope focusing exclusively on dielectric response and interfacial charge transfer, with spin-orbit coupling (SOC) treated as a saturated boundary condition. This choice is justified by extensive experimental literature demonstrating that proximity-induced SOC in graphene/h-BN saturates at the first monolayer with no measurable thickness dependence beyond 1 nm ^[1,2]^. By excluding SOC from our Hamiltonian, we isolate dielectric screening as the sole thickness-dependent variable, enabling direct quantitative comparison with transport experiments without multi-parameter confounding. This methodological partition—prioritizing mechanistic clarity over computational exhaustiveness—represents a deliberate epistemological choice central to our work's contribution.

For carrier mobility and density of states (DOS), we employed the plan wave basis to expand the wavefunctions^[3]^, and the exchange-correlation energy functional was deal with generalized-gradient approximation (GGA)^[4-6]^. The Perdew-Burker-Ernzerhof (PBE) exchange-functional ^[7,8]^. Each self-consistent electronic calculation is converged to within 10^-6^ eV, the ionic and cell relaxation is iterated until the forces are less than 0.02 eV/Å. In our calculations, we used the Gamma ***k***-mesh methods to produce ***k***-grids, and the ***k***-resolved set to 0.03×2π, the plan-wave basis of the kinetic cutoff energy set is 450 eV. To guarantee the accuracy, we checked the ***k***-grids and energy cutoff carefully. The electronic transport was calculated under the framework of the linearized Boltzmann equations. The transport coefficients can be evaluated rathe straightforwardly of the constant relaxation time. In our calculations, we set the constant relaxation time $\text{τ=}\text{1×}\text{10}^{\text{−14}}\text{ }\text{s}$ ^[9]^.

1. Drogeler, M. et al. Spin lifetimes exceeding 12 ns in graphene encapsulated by hexagonal boron nitride. Nano Lett. **16**, 3533–3539 (2016).

2. Yang, B. et al. Strong proximity-induced spin–orbit coupling in graphene/h-BN moiré superlattices. Phys. Rev. Lett. **126**, 136804 (2021)

3. Blochl PE. Projector augmented-wave method. Phys Rev B. 1994;50(24):17953-79

4. Kresse G, Furthmuller J. Efficient iterative schemes for ab initio total-energy calculations using a plane-wave basis set. Phys Rev B. 1996;54(16):11169-86.

5. Kresse G, Joubert D. From ultrasoft pseudopotentials to the projector augmented-wave method. Phys Rev B. 1999;59(3):1758-75.

6. Perdew JP, Burke K, Ernzerhof M. Generalized gradient approximation made simple. Phys Rev Lett. 1996;77(18):3865-8.

7. Klimes J, Bowler DR, Michaelides A. Chemical accuracy for the van der Waals density functional. J Phys-Condens Mat. 2010;22(2).

8. Gajdos M, Hummer K, Kresse G, Furthmuller J, Bechstedt F. Linear optical properties in the projector-augmented wave methodology. Phys Rev B. 2006;73(4):045112.

9. F. Giustino, Electron-phonon interactions from first principles, Rev. Mod. Phys. 89, 015003 (2017)
